# Supplementary material for: The causal role of circulating inflammatory markers in osteoporosis: a bidirectional Mendelian randomized study
Source: Front Immunol. 2024 Jul 18;15:1412298. doi: 10.3389/fimmu.2024.1412298 (PMC11291241; doi:10.3389/fimmu.2024.1412298)
Supplement: Supplementary file 1 [file DataSheet_1.zip › supplementary material/OSTEOPOROSIS-CIM/GCST90274770.tsv.gz_leave_one_out.pdf]

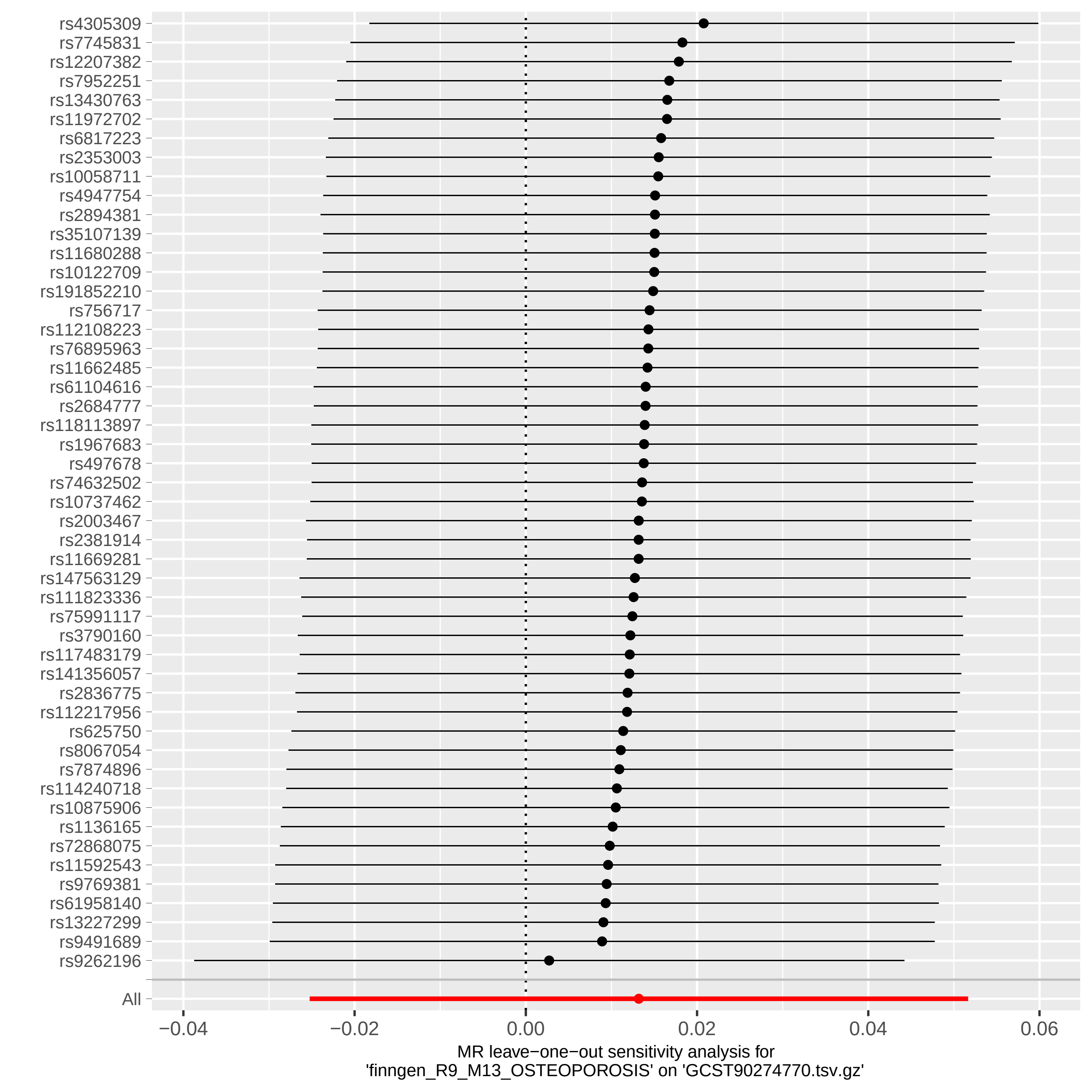

rs4305309
rs7745831
rs12207382
rs7952251
rs13430763
rs11972702
rs6817223
rs2353003
rs10058711
rs4947754
rs2894381
rs35107139
rs11680288
rs10122709
rs191852210
rs756717
rs112108223
rs76895963
rs11662485
rs61104616
rs2684777
rs118113897
rs1967683
rs497678
rs74632502
rs10737462
rs2003467
rs2381914
rs11669281
rs147563129
rs111823336
rs75991117
rs3790160
rs117483179
rs141356057
rs2836775
rs112217956
rs625750
rs8067054
rs7874896
rs114240718
rs10875906
rs1136165
rs72868075
rs11592543
rs9769381
rs61958140
rs13227299
rs9491689
rs9262196
All
−0.04
−0.02
0.00
0.02
0.04
0.06
MR leave−one−out sensitivity analysis for
'finngen_R9_M13_OSTEOPOROSIS' on 'GCST90274770.tsv.gz'
